# Supplementary material for: Availability and utilization of sexual and reproductive health services among adolescents of Godawari Municipality, Nepal: A cross-sectional study
Source: PLOS Glob Public Health. 2025 Jul 2;5(7):e0003282. doi: 10.1371/journal.pgph.0003282 (PMC12220999; doi:10.1371/journal.pgph.0003282)
Supplement: Attachment — Submitted filename: Review-Plos Global PH.pdf [file pgph.0003282.s003.pdf]

1. Keep keywords in alphabetical order in abstract.
2. Is your study method descriptive cross sectional? Can the inferential statistical analysis be done in descriptive studies?
3. Elaborate the sample size calculation in detail. How did you calculate the value of n as 416?
4. Sample size has been mentioned different through the study. The calculated was 416 but in table 1 variable age n is 413, n in level of education, n in relationship status is 412. In table 2 too, n is different for each variable.(perceived need of separate service-415 distance to health education-322, necessity of convenient opening hours of institution-414, Perceived Community support-415, Perceived Effect of sex of service provider-413. Why is the difference?
5. In Parents support in use of services (n=413) but n in Conversation with parents about ASRH is 416. Why is the difference? Is there error in data collection or during data management?
6. There are errors in giving asterisk (*) in table 1 for association p value. Check properly and footnote might be necessary for the meaning of asterisk (*).
7. In line number 162, sentence is not clear. The meaning of until now is not clear. Re-structure the sentence.
8. In Table 4, mention the meaning of * (multiple response).
9. Check Table 5 properly for clearly view of contents. Bullet might not be necessary.
10. Conclusion in the manuscript has been presented as summary of Results. Needs revision in conclusion part.
