# Supplementary material for: Availability and utilization of sexual and reproductive health services among adolescents of Godawari Municipality, Nepal: A cross-sectional study
Source: PLOS Glob Public Health. 2025 Jul 2;5(7):e0003282. doi: 10.1371/journal.pgph.0003282 (PMC12220999; doi:10.1371/journal.pgph.0003282)
Supplement: Attachment — Submitted filename: renamed_9c7f5.docx [file pgph.0003282.s005.docx]

**Response to Reviewers:**

PGPH-D-24-00995: Availability and Utilization of Sexual and Reproductive Health Services among Adolescents of Godawari Municipality, Nepal: a Cross sectional Study

Dear Editor and Reviewers,

PLOS Global Public Health,

Greetings from Nepal,

I would like to extend my sincere gratitude to the editorial and review team for the constructive suggestions and comments in our manuscript. This has undoubtedly helped us shape our manuscript to meet the standards of the global scientific arena. Your valuable suggestions and comments were very meaningful to us. We have gone through all of the comments and have tried our best to rectify them. Our responses are as follows;

**Response to general comments:**

Comment (C):

Response (R): **C-**1. Does this manuscript meet PLOS Global Public Health’s [publication criteria](https://journals.plos.org/globalpublichealth/s/criteria-for-publication)? Is the manuscript technically sound, and do the data support the conclusions? The manuscript must describe methodologically and ethically rigorous research with conclusions that are appropriately drawn based on the data presented.

Reviewer #1: Partly

Reviewer #2: Partly

R-1-The manuscript was reviewed and revised as per the suggestions of reviewer’s including the methodology and conclusion.

C-2. Has the statistical analysis been performed appropriately and rigorously?

**Reviewer #1: No**

Reviewer #2: Yes

R-2. The statistical analysis was revised.

C-3. Have the authors made all data underlying the findings in their manuscript fully available (please refer to the Data Availability Statement at the start of the manuscript PDF file)?

The [PLOS Data policy](https://journals.plos.org/globalpublichealth/s/data-availability) requires authors to make all data underlying the findings described in their manuscript fully available without restriction, with rare exception. The data should be provided as part of the manuscript or its supporting information, or deposited to a public repository. For example, in addition to summary statistics, the data points behind means, medians and variance measures should be available. If there are restrictions on publicly sharing data—e.g. participant privacy or use of data from a third party—those must be specified.

**Reviewer #1: No**

**Reviewer #2: No**

R-3.Data have been uploaded as supplementary files.

C-4. Is the manuscript presented in an intelligible fashion and written in standard English?

PLOS Global Public Health does not copyedit accepted manuscripts, so the language in submitted articles must be clear, correct, and unambiguous. Any typographical or grammatical errors should be corrected at revision, so please note any specific errors here.

**Reviewer #1: Yes**

Reviewer #2: No

R-4. Grammatical errors and other mistakes have been identified and corrected throughout the document as applicable. A colleague who is an expert in the English language was consulted for doing so.

5. Review Comments to the Author

Please use the space provided to explain your answers to the questions above. You may also include additional comments for the author, including concerns about dual publication, research ethics, or publication ethics. (Please upload your review as an attachment if it exceeds 20,000 characters)

**Response to the comments of Reviewer 1:**

C-1. Keep keywords in alphabetical order in abstract.

R-1: The keywords have been kept in alphabetical order in the abstract.

*Adolescents; Availability, Nepal, Sexual and Reproductive Health Services; Utilization*

C-2. Is your study method descriptive cross sectional? Can the inferential statistical analysis be done in descriptive studies?

R-2. Basically, our study is a cross sectional study aimed at determining the status of the availability and utilization of ASRHS. Since we had the study population categorised on the basis of SRHS utilization as a) SRHS utilized and b) SRHS not utilized, it allowed us to make comparisons between those two groups of respondents. Therefore, we further analysed using inferential statistics to estimate the risk (causal effect based on the strength of association) between different independent variables and dependent variables.

The term descriptive has been omitted from the materials and method section.

C-3. Elaborate the sample size calculation in detail. How did you calculate the value of n as 416?

R-3. The sample size was determined using the formula for infinite population developed by Cochran which is

n=z²p(1-p) /d²

Where, n= desired sample size

Z = Constant which is 1.96 at 95% CI, p = 44% = 0.4413 ^16^

d (allowable error) = 5% = 0.05

n=z²p(1-p) /d²

=1.962*0.44*0.56/ 0.052

=378.4

Taking 10% of the sample size as non-response rate, the final sample size was; =378.4 + 10% of 378.4 = 416.2 ~ 416

C-4. Sample size has been mentioned different through the study. The calculated was 416 but in table 1 variable age n is 413, n in level of education, n in relationship status is 412.

In table 2 too, n is different for each variable.( perceived need of separate service-415 distance to health education-322, necessity of convenient opening hours of institution 414, Perceived Community support-415, Perceived Effect of sex of service provider-413.

Why is the difference?

R-4: The data was collected using self-administered questionnaire. Also, the participants rights to autonomy, the right to withdraw from the study at any time and the right to reject any questions during data collection were respected. Therefore, we did not force our participants to answer all the questions. It was a matter of voluntary contribution from the participants. This has led to the issue of missing data in our study. Therefore, the total number of responses in different variables is different.

C-5. In Parents support the use of services (n=413) but n in Conversation with parents about ASRH is 416. Why is the difference? Is there error in data collection or during data management?

R-5. In addition to the response 4 the difference may be due to the absence of parents to support them physically. Moreover, adolescents can talk to their parents even though they are not together with them.

C-6. There are errors in giving asterisk (*) in table 1 for association p value. Check properly and footnote might be necessary for the meaning of asterisk (*).

R-6. The asterisk (*) sign changed to (++) in table 1 which denoted the chi-square value obtained from “Fisher’s exact test.” So, for clarity, a footnote has been added in table 1 and others, wherever applicable.

C-7. In line number 162, sentence is not clear. The meaning of until now is not clear. Restructure the sentence.

Almost half of the respondents reported that they had felt the need for ASRH services (48.56%) until now.

R-7. The sentence has been revised as:

Almost half of the respondents felt the need for ASRH services (48.56%).

C-8. In Table 4, mention the meaning of * (multiple response).

R-8. Information has been added in the footnote as:

¶ Multiple responses; therefore, the total percentage exceeds 100%.

C-9. Check Table 5 properly for clearly view of contents. Bullet might not be necessary.

R-9. Required revisions are made accordingly.

10. Conclusion in the manuscript has been presented as summary of Results. Needs revision in conclusion part.

**The conclusion part has been thoroughly revised as;**

Most of the participants were 15-17 years old, female, and studying in class 12. Very few were married or in a relationship. Approximately one-third of the participants engaged in discussion with their parents about various aspects of ASRH. Many knew about any health institutions that provided ASRH services in their area. For the majority of respondents, the nearest health institution was less than 30 minutes walking distance from their home. Only a few had heard about AFHS. Although the majority felt the need for ASRH services, they were still unsure about using services in the future.

The utilization of ASRH services among participants was very low. The poor utilization of SRH services was due to the inability of adolescents to identify their sexual and reproductive health needs. Some of the reasons were being uninterested in using such services, being ashamed to ask for services, and poor availability of AF health institutions and services. As this study clearly revealed that adolescents lack sufficient knowledge and a positive attitude towards using ASRH services. Therefore, to enhance their understanding and use of ASRH services, we must prioritize providing them with an adequate access to information and health services. Also, establishing adolescents-friendly infrastructure and environments along with the availability of trained health personnel to provide those services can guarantee the optimum utilization of ASRH services as per the need.

**Response to the comments of Reviewer 2:**

This is an impressive study, and the authors wanted to highlight the availability and utilization of reproductive health services among youths of classes 11 and 12.

The methodology needs further clarification and justification, such as

C-1. why government schools, not private schools, and

R-1. The study was conducted in Godawari Municipality, Lalitpur District, Nepal. Godawari Municipality was chosen purposively because it represents semi-urban areas and was formed by merging the former 12 Village Development Committees (VDCs). The study is delimited to government schools to illustrate the situation of semi urban population in resource limited setting. Private school students perform better than public school students, mostly in Nepal. If both public and private schools were selected, there could be a positive private school effect in the findings of this study. [(13)](https://www.researchgate.net/publication/271857200_Public_and_private_school_performance_in_Nepal_an_analysis_using_the_SLC_examination) In addition, the public schools lack resources to get access to information and opportunities, which may differ from the situation for the students of private schools, who may have better opportunities to access information and health care services. (14)

C-2. Why two months only.

R-2. Information collection was completed in 3 months; among these 3 months, two months were for preparation. The information was collected in January 2022 only, which was possible because of the use of self-administered questionnaire.

C-3. It is expected that seasonal variations will be a major factor in the accessibility and utilization of ASRH.

R-3. Even though information was collected within a limited time period, the study was about their previous experiences, which was not limited to the study period only.

C-4. What is the likelihood of these youths using the nearby health facilities in the municipality near the capital city?

R-4. The local health institutions were studied to figure out the providers' perspectives only; however the utilization of ASRHS by the study participants was not limited to local health institutions. They reported the services they used from anywhere.

C-5 language needs to be revised and corrected.

R-5. Grammatical errors and other mistakes have been identified and corrected throughout the document as applicable. A colleague who is an expert in the English language was consulted for doing so.

C-6. The research did not show any impact of COVID and lockdown on access to and use of ASRH from the user and provider side

R-6. The study was conducted after COVID-19. The schools already resumed all of the physical classes normally as pre-COVID-19 period but with mandatory use of mask. Therefore, this study did not have any impact of COVID-19 restrictions. However, the effects of COVID-19 on the utilization of health services by adolescents were not measured.

We attempted to rectify all comments and suggestions that had been raised by the reviewers. We hope that all the concerns have been addressed. We are very grateful for your time and feedback.

Thank you very much.

With Best Regards

Laxmi Gautam
