# Supplementary material for: Availability and utilization of sexual and reproductive health services among adolescents of Godawari Municipality, Nepal: A cross-sectional study
Source: PLOS Glob Public Health. 2025 Jul 2;5(7):e0003282. doi: 10.1371/journal.pgph.0003282 (PMC12220999; doi:10.1371/journal.pgph.0003282)
Supplement: Attachment — Submitted filename: renamed_03c0c.docx [file pgph.0003282.s006.docx]

**Response to Reviewers:**

**PGPH-D-24-00995R1**: Availability and Utilization of Sexual and Reproductive Health Services among Adolescents of Godawari Municipality, Nepal: a Cross sectional Study

Dear Editor and Reviewers,

PLOS Global Public Health,

Greetings from Nepal,

I would like to extend my sincere gratitude to the editorial and review team for the constructive suggestions and comments in our manuscript. This has undoubtedly helped us shape our manuscript to meet the standards of the global scientific arena. Your valuable suggestions and comments were very meaningful to us. We have gone through all of the comments and have tried our best to rectify them. Our responses are as follows;

**Response to general comments:**

Comment (C):

Response (R):

**1**. If the authors have adequately addressed your comments raised in a previous round of review and you feel that this manuscript is now acceptable for publication, you may indicate that here to bypass the “Comments to the Author” section, enter your conflict of interest statement in the “Confidential to Editor” section, and submit your "Accept" recommendation.

Reviewer #2: (No Response)

Response: NA

**2**. Does this manuscript meet PLOS Global Public Health’s [publication criteria](https://journals.plos.org/globalpublichealth/s/criteria-for-publication)? Is the manuscript technically sound, and do the data support the conclusions? The manuscript must describe methodologically and ethically rigorous research with conclusions that are appropriately drawn based on the data presented.

Reviewer #2: Partly

Response: The manuscript was reviewed and revised as per the suggestions of reviewer’s including the methodology and conclusion.

3. Has the statistical analysis been performed appropriately and rigorously?

Reviewer #2: **Yes**

Response: Thank you for your appreciation.

4. Have the authors made all data underlying the findings in their manuscript fully available (please refer to the Data Availability Statement at the start of the manuscript PDF file)?

The [PLOS Data policy](https://journals.plos.org/globalpublichealth/s/data-availability) requires authors to make all data underlying the findings described in their manuscript fully available without restriction, with rare exception. The data should be provided as part of the manuscript or its supporting information, or deposited to a public repository. For example, in addition to summary statistics, the data points behind means, medians and variance measures should be available. If there are restrictions on publicly sharing data—e.g. participant privacy or use of data from a third party—those must be specified.

**Reviewer #2: Yes**

Response: Thank you

5. Is the manuscript presented in an intelligible fashion and written in Standard English?
PLOS Global Public Health does not copyedit accepted manuscripts, so the language in submitted articles must be clear, correct, and unambiguous. Any typographical or grammatical errors should be corrected at revision, so please note any specific errors here.

Reviewer #2: No

Response: Grammatical errors and other mistakes have been identified and corrected throughout the document as applicable. A colleague who is an expert in the English language was consulted for doing so.

6. Review Comments to the Author

Please use the space provided to explain your answers to the questions above. You may also include additional comments for the author, including concerns about dual publication, research ethics, or publication ethics. (Please upload your review as an attachment if it exceeds 20,000 characters)

**Response to the comments of Reviewer 2:**

C-1. Study is only one directional and it is not unique to many studies conducted or published in the peer reviewed article.
In my view, exclusion of the private school students may create potential bias while the study acknowledged that private school student often has better access to resources. This is a limitation that it only reflects the one subset of the adolescent of Nepal represented from well to do societies and high income group, if I am not wrong.

R-1. Thank you for your valuable concern. Indeed, the exclusion of private schools may create potential bias. However, the study was conducted in Godawari Municipality, Lalitpur District, Nepal. Godawari Municipality was chosen purposively because it represents semi-urban areas and was formed by merging the former 12 Village Development Committees (VDCs). The study is delimited to government schools to illustrate the situation of semi urban population in resource limited setting. Private school students perform better than public school students, mostly in Nepal. If both public and private schools were selected, there could be a positive private school effect in the findings of this study as highlighted by the literature (reference# 13). In addition, the public schools lack resources to get access to information and opportunities, which may differ from the situation for the students of private schools, who may have better opportunities to access information and health care services (reference# 14).

(information provided in methods section)

C-2: The other weakness is the qualitative sample size in the study. It is quite limited (only three) and it is purposive selection of health care provider near the school, which shall be skewed towards more accessible or better equipped health facilities.
I think, it would have been more informative if the research has taken the qualitative information from the student as well to see from their perspective as well.

R-2. The respondents for qualitative study was chosen based on the selection of health care provider near the schools. For quantitative study, we selected 3 government schools randomly (from a total of 10 government schools). The health facility related information we receive may be assumed same if we take one or more than one respondents from the same health facilities. Therefore, we thought of limiting the qualitative participants to one per health institution with close proximity to the selected schools. And moreover, since the study site is semi (or peri) urban area, the health care accessed was assumed to have been same irrespective of the schools selected. The local health institutions were studied to figure out the providers' perspectives only; however, the utilization of ASRHS by the study participants was not limited to local health institutions. They reported the services they used from anywhere.

(information provided in method section)

C-3: This study showed a correlation between the parental discussion and likelihood of using the services but fail to mention the reason behind it. The author tried to compare the parental communications findings with Ethiopia but didn’t mention why these countries would be appropriate to compare given their different societal norms and cultural differences. I think, poverty should not only be the case for comparisons.

R-3: Parental communication is often regarded as the protective factor for adolescent health. They are potentially an important source of ARSH related information to their children and can greatly influence children attitudes and behaviors towards their health including ARSH. Parental discussion promotes likelihood of preventing from adopting unhealthy ARSH behavior and practices leading to promotion of healthy lifestyles. (24) Students who have communicates SRH related concerns with their parents plays a significant role in promoting adolescent intentions for healthy ASRH behaviors and practices.(25) Another study conducted in India also prevailed positive effects of parental communication in the utilization of ASRHS mentioning that decreasing adolescent-parents communication related to sexual issues of was associated with nonutilization of ASRH services.(26) Additionally, parental communication leads on reducing risky sexual behavior as well as utilize adolescent friendly services related to ARSH. (24) These findings from similar cultural context are supportive findings to prevail the importance of parents and adolescents communication in the issues related to sexual and reproductive health.

(Information added in discussion section)

C-4: The reader might be more interested to see beyond the basic explanations like stigma and lack of information’s (13.22%). But failed to mention the structural issues such as health system, lack of human resources etc. that country like Nepal usually suffers from.
I think, if this information were captured, we could have more information about the barriers to utilizations of ASRH.

R-4: The reason for not utilizing ARSH services were mostly personal and health system related factors. Some of personal factors were: need was not perceived (60%): not interested in such services (38%); ashamed to ask for services (26%), etc. Likewise, health system related factors includes inconvenient opening hours (6%); access to health facility (4%); health work force related (8%), finance related (3%), etc.

(Information is added in discussion)

C-5. The research did not show any impact of COVID and lockdown on access to and use of ASRH from the user and provider side

R-5. The study was conducted after COVID-19. The schools already resumed all of the physical classes normally as pre-COVID-19 period but with mandatory use of mask. Therefore, this study did not have any impact of COVID-19 restrictions. However, the effects of COVID-19 on the utilization of health services by adolescents were not measured which was the limitation of this study.

(Information is provided in discussion section as limitation)

We attempted to rectify all comments and suggestions that had been raised by the reviewers. We hope that all the concerns have been addressed. We are very grateful for your time and feedback.

Thank you very much.

With Best Regards

Laxmi Gautam
