# Supplementary material for: Availability and utilization of sexual and reproductive health services among adolescents of Godawari Municipality, Nepal: A cross-sectional study
Source: PLOS Glob Public Health. 2025 Jul 2;5(7):e0003282. doi: 10.1371/journal.pgph.0003282 (PMC12220999; doi:10.1371/journal.pgph.0003282)
Supplement: Attachment — Submitted filename: Response to Reviewers 4.docx [file pgph.0003282.s008.docx]

**Response to Reviewers:**

**PGPH-D-24-00995R1**: Availability and Utilization of Sexual and Reproductive Health Services among Adolescents of Godawari Municipality, Nepal: a Cross sectional Study

Dear Editor and Reviewers,

PLOS Global Public Health,

Greetings from Nepal,

I would like to extend my sincere gratitude to the editorial and review team for the constructive suggestions and comments in our manuscript. This has undoubtedly helped us shape our manuscript to meet the standards of the global scientific arena. Your valuable suggestions and comments were very meaningful to us. We have gone through the comment and have tried our best to rectify them.

**Response to the comment:**

Reviewer #1: I am not satisfied with your answer. As per you, "The total 416 samples included the 378 actual sample sizes and added 10% of the sample size. "There are some missing data in the study, but the total sample size of 416 included the 10%
non-response rate of the actual sample size of 378, so there should be no problem with the validity of the study."

1. Do you mean missing data and non-respondents are same? Can the data which has been missed be referred as non-respondents?

Response: As I already mentioned (which of course, you are not satisfied by the answer), the final sample size (416) included the non-response rate. The non-response rate was considered anticipating the sudden-withdrawal of the participants during the process of ongoing data collection or respondent’s unwillingness to answer, or respondents unaware about the answer of the questions, leading to missing data.

Here, addition of non-response of the information from the participants who take part in the data collection, but does not complete the response, thus leading to missing data. Therefore, non-response used in this study should not be made confused with non-participants, who rejects to participate in the study.

2. Next the data missed may be of different respondents for different variable. Can you conclude saying missing data as non-respondents on that case? You need to consult a statistician before giving any judgment as this manuscript is for the publication in a renowned journal.

Response: In this study, the high non-response was observed for the variable – Distance to Health Institution, only leading to missing data. As I consulted with the statistician, he told me that it can happen usually for one variable to have more non-response. The reason being most of the respondents might find it difficult or hesitate to answer because of the special nature of adolescents and some conditions might not be applicable to all of them. In our case, though the variable to measure was not complex, it was unexpected that many respondents did not respond to this variable.
3. In Table 2: Need and External Environmental factors of ASRHS, the sample size of variable 'utilization
Non Distance to health institution' is 322 which is much below 378. How can you ensure the validity on that case?

Response: As we already mentioned above, the missing data was due to high-non response to that variable. As I consulted with the statistician, they responded me that since the variable was non-significant in bivariate analysis and not included in the multi-variate analysis, the final sample size for the multivariate analysis remained unchanged, and highest sample size was thought to be considered. Hence, we believe that it does not affect the validity of our analysis and research as a whole.

The following article was referred for dealing with missing data and non-response.

Ardalan Mirzaei, Stephen R. Carter, Asad E. Patanwala, Carl R. Schneider,Missing data in surveys: Key concepts, approaches, and applications,Research in Social and Administrative Pharmacy, Volume 18, Issue 2, 2022, Pages 2308-2316, ISSN 1551-7411, <https://doi.org/10.1016/j.sapharm.2021.03.009>.

We hope that your concern has been addressed. We are very grateful for your time and feedback.

Thank you very much.

With Best Regards

Laxmi Gautam
